# Novel Antibiotic Testing Approaches Reveal Reduced Antibiotic Efficacy Against Shiga Toxin-Producing *Escherichia coli* O157:H7 Under Simulated Microgravity

**DOI:** 10.3389/fmicb.2018.03214

**Published:** 2018-12-21

**Authors:** Hye Won Kim, Min Suk Rhee

**Affiliations:** Department of Biotechnology, College of Life Sciences and Biotechnology, Korea University, Seoul, South Korea

**Keywords:** *Escherichia coli* O157:H7, space environment, antibiotic test, Shiga toxin gene, cellular mechanism

## Abstract

As a foodborne and environmental pathogen, Shiga toxin-producing *Escherichia coli* O157:H7 could pose a health threat to immunocompromised astronauts during a space mission. In this study, novel approaches, including real-time testing and direct evaluation of resistance mechanisms, were used to evaluate antibiotic efficacy against *E. coli* O157:H7 under low-shear modeled microgravity (LSMMG) produced using a rotary cell culture system. When compared with normal gravity (NG), bacterial growth was increased under LSMMG in the presence of sub-inhibitory nalidixic acid concentrations and there was an accompanying up-regulation of stress-related genes. LSMMG also induced transcriptional changes of the virulence genes *stx1* and *stx2*, highlighting the potential risk of inappropriate antibiotic use during a spaceflight. The degree of bacterial cell damage induced by the antibiotics was reduced under LSMMG, suggesting low induction of reactive oxygen species. Efflux pumps were also shown to play an important role in these responses. Increased cell filamentation was observed under LSMMG upon ampicillin treatment, possibly reflecting a protective mechanism against exposure to antibiotics. These observations indicate that, in the presence of antibiotics, the survival of *E. coli* O157:H7 is greater under LSMMG than under NG, indicating that antibiotic therapies may need to be adjusted during space missions.

## Introduction

On-board infections are of concern to those seeking to promote manned space exploration (Taylor and Sommer, 2005). Increased bacterial capacity to cause disease (Wilson et al., 2007; Crabbé et al., 2011) and the weakening of the immune system in space (Crucian et al., 2008), especially, could give rise to conditions favoring bacterial infections. Indeed, evidence exists for urinary tract infections among the crew of the Apollo 13 spaceflight mission (Taylor, 1974), and in-flight cross-contamination of the upper respiratory tract (Decelle and Taylor, 1976), conjunctivitis, and acute respiratory and dental infections among astronauts living on the Russian space station Mir (Ball and Evans, 2001). The transmission of antibiotic-resistant strains from one astronaut to another on the Soviet and International Space Stations has also been reported (Ilyin, 2005).

As incidents of bacterial infections and the transfer of antibiotic-resistant bacteria in space have been documented, the susceptibility of bacteria to antibiotics under microgravity conditions has been studied for several decades. Increased bacterial resistance to antibiotics has been reported by various researchers (Tixador et al., 1985a; Lapchine et al., 1986; Juergensmeyer et al., 1999) and evidence exists for the reduction of bioavailability of orally-administered drugs in space (Tietze and Putcha, 1994). The major causes of these responses are, however, not yet clear. Therefore, clarification of the efficacy of antibiotics in space or a space-like environment is a critical issue that needs to be carefully considered.

Investigations into the effect of microgravity on microorganisms during real spaceflight are limited by flight opportunities, financial costs, the need for specialized equipment in the spacecraft or space station, and astronauts’ time (Nickerson et al., 2004). As an alternative, a ground-based spaceflight analog bioreactor, the High-Aspect Ratio Vessel (HARV; Synthecon, Houston, TX, United States), was developed at the Johnson Space Center/National Aeronautics and Space Administration. It produces a low-shear microgravity environment similar to that found in space. The simulated low gravity environment is generated by the rotation of the HARV, which continuously maintains the gravitational vector experienced by the bacterial cells to near-zero (Valluri and Gonda, 2006).

In the current study, an attempt was made to assess antibiotic efficacy under simulated microgravity, typical of the space environment, against a pathogenic bacterium of significant public health impact, *Escherichia coli* O157:H7. This is a foodborne and environmental pathogen that can cause life-threatening human disease even when only a small number of cells are consumed (Phillips, 1999). Antibiotic-resistant strains of this serotype pose a risk to food safety and public health (Schroeder et al., 2002). *E. coli* O157:H7 strains can produce one or both of the two types of Shiga toxin (Stx1 and Stx2) encoded by the *stx* genes. The use of antibiotics to treat infections may lead to unexpected side effects such as Shiga toxin induction (Kimmitt et al., 2000). The strain studied here, ATCC 43895, was originally isolated from ground beef linked to a large outbreak in the United States in 1982 (Riley et al., 1983) and harbors both *stx1* and *stx2* (Bürk et al., 2003).

The main goals of this study were to determine the efficacy of antibiotics that target different biological molecules [ampicillin (AM) targeting peptidoglycan, gentamicin (GM) targeting the ribosome, and nalidixic acid (NA) targeting DNA] against *E. coli* O157:H7 under simulated microgravity using novel approaches overcoming the limits of conventional antibiotic susceptibility testing, and to determine the mechanisms responsible for resistance. Growth-monitoring and inactivation assays were conducted since they reveal the response of bacteria under simulated microgravity over time. Cellular responses to antibiotics and the effects of antibiotic treatments were evaluated on the basis of (i) molecular genetic analysis of the antibiotic stress response (*rpoS, oxyR*, and *soxR*) (Lushchak, 2001; Matin, 2009) and virulence-related genes (*stx1* and *stx2*), (ii) flow cytometry analysis of cell damage (Saint-Ruf et al., 2016), (iii) an efflux accumulation assay using ethidium bromide (EtBr), a common substrate of efflux pumps of the *Enterobacteriaceae* (Schumacher et al., 2007), and (iv) direct observation of morphological changes by microscopy.

## Materials and Methods

### Bacterial Strains and Growth Media

All studies were performed using *E. coli* O157:H7 ATCC 43895 from the American Type Culture Collection. This isolate, also referred to as CDC EDL933, was originally isolated from ground beef linked to a large outbreak of foodborne disease in the United States in 1982 (Riley et al., 1983), and it harbors the *stx1* and *stx2* genes (Bürk et al., 2003). Bacterial cells were stored at -80°C in a medium containing 20% glycerol. When required, the cells were grown overnight in Muller-Hinton (MH) broth (Difco, Sparks, MD, United States) at 37°C in a shaking incubator set at 225 rpm (VS-8480S, Vision Scientific, Co., Ltd., Seoul, South Korea). The initial optical density at 660 nm of stationary phase cultures was measured using a SmartSpec^TM^ Plus spectrophotometer (Bio-Rad, Hercules, CA, United States).

### Equipment Used for LSMMG and NG Experiments

The Rotary Cell Culture System^TM^ (RCCS^TM^; Synthecon) and a 50 ml HARV (Synthecon) were used to generate low-shear modeled microgravity (LSMMG, spaceflight analog conditions) and normal gravity (NG, Earth conditions) in the laboratory, as described in previous studies (Kim et al., 2014; Kim and Rhee, 2016). The HARV apparatus was filled with the appropriate overnight cultures, with zero headspace and no bubbles, and maintained in a constant state of suspension. The vessels were rotated at 25 rpm around a horizontal axis for LSMMG, with the hydrodynamic forces, including centrifugal, Coriolis, and shear forces, offsetting the gravitational force in the bioreactor. The counterpart NG conditions were obtained by rotating the vessels around a vertical axis. All incubations were performed at 37°C, and a gas-permeable membrane on the back of HARV allowed air exchange during incubation.

### Susceptibility Testing by the Disk Diffusion Method

Antibiotic susceptibility was tested by the disk diffusion method according to the recommendations of the Clinical and Laboratory Standards Institute (CLSI, 2012). The surface of MH agar (Difco) was inoculated with a swab dipped in the cell suspension with a turbidity adjusted to 0.5 McFarland standard. Commercial antibiotic susceptibility disks (Oxoid, Basingstoke, United Kingdom), including AM (10 μg), GM (10 μg), and NA (30 μg), were placed onto the surfaces of the inoculated plates and the plates were then incubated at 37°C for 18 h. The diameter of the inhibition zone was measured and the antibiotic susceptibility was classified as resistant (R), intermediate (I), or susceptible (S), based on the manufacturer’s instructions. All experiments were repeated six times.

### MIC Determination by the Broth Microdilution Assay

Antibiotic solutions (Sigma-Aldrich, St. Louis, MO, United States) were freshly prepared before each experiment. The minimum inhibitory concentration (MIC) values for *E. coli* O157:H7 cells cultured under LSMMG and NG were determined by the method currently recommended by CLSI (Watts et al., 2008) in sterile, 96-well, U-bottomed plates. Briefly, each microdilution well-containing 100 μl of the corresponding twofold antibiotic dilution was inoculated with 100 μl of a cell suspension (final concentrations of ca. 5.0 × 10^5^ CFU/ml). The microdilution trays were incubated at 37°C for 18 h, and the MIC was defined as the lowest concentration of antibiotic for which no visible cell growth was observed. In each case, cell suspensions inoculated in the absence of antibiotics served as the positive control. The MIC values determined (Table 1) were used to calculate antibiotic concentrations corresponding to 1/2 × MIC and 4 × MIC. All experiments were repeated in triplicate.

**Table 1:** The susceptibility and Minimum Inhibitory Concentrations (MIC) of the tested antibiotics against *E. coli* O157:H7 ATCC 43895 cultured under LSMMG and NG in MH broth for 24 h.

Biological effect	Class	Antibiotic	Primary target	Diameter of inhibition zone, mm (antibiotic susceptibility)		MIC (mg/L)	
				LSMMG	NG	LSMMG	NG
Cell wall synthesis inhibitor	β-Lactam	Ampicillin (AM)	Penicillin-binding proteins	17.0 ± 1.4 (S^b^)	17.8 ± 1.3 (S)	1.0	1.0
Protein synthesis inhibitor	Aminoglycoside	Gentamycin (GM)	30S^a^ ribosomal subunit	21.8 ± 1.3 (S)	21.5 ± 1.7 (S)	0.5	0.5
DNA synthesis inhibitor	Quinolone	Nalidixic acid (NA)	Topoisomerase II Topoisomerase IV	22.8 ± 0.5 (S)	22.8 ± 1.9 (S)	4.0	4.0

*Mean ± SD; *n* = 6 per group; ^*a*^S = A svedgerg unit; ^*b*^S = susceptible.*

### Growth Kinetics at Sub-inhibitory Antimicrobial Concentrations

Overnight cultures were diluted in MH broth supplemented with sub-inhibitory concentrations of each antibiotic (1/2 × MIC) and then incubated under LSMMG or NG, as described above. A sterile 5 ml syringe was used to remove 500 μl samples through a port on the front plate of the HARV after 0, 3, 6, 12, 24, and 48 h of incubation. The zero headspace within the HARV was maintained by adding an equivalent volume of sterile 0.85% saline. Bacterial growth was monitored by measuring the OD_600_ (Bio-Rad). The numbers of viable cells were determined using the plate-counting method. Briefly, the cells were serially diluted 10-fold in sterile 0.85% saline and spread-plated onto MH agar, and the colonies were counted after incubation at 37°C for 24 h. All experiments were repeated at least six times.

### RNA Extraction and cDNA Synthesis

Total RNA was extracted from cells that had been cultured in the MH broth for 24 h under LSMMG and NG using TRIzol^®^ (Invitrogen, Grand Island, NY, United States), following the manufacturer’s instructions. The concentration of total RNA was determined using an ND-1000 spectrophotometer (NanoDrop Technologies, Wilmington, DE, United States). The quality of RNA in a sample was deemed to be acceptable if the A_260/280_ ratio was 1.9–2.1. The sample RNA was reverse-transcribed using the high-capacity cDNA reverse transcription kit (Applied Biosystems, Foster City, CA, United States) according to the manufacturer’s instructions. Briefly, 2.0 μg RNA was reverse-transcribed in a reaction containing 10 × random primers, 25 × dNTP mix (100 mM), 10 × buffer, 1.0 μl MultiScribe^TM^ reverse transcriptase, and 1.0 μl RNase inhibitor, with a total reaction volume of 20 μl. cDNA synthesis was performed in a thermo-cycler (Thermo Fisher Scientific, Rockford, IL, United States) with the following cycling conditions: 25°C for 10 min, 37°C for 120 min, and 85°C for 5 min, followed by 4°C to stop the reaction.

### qRT-PCR for the Determination of the Stress and Virulence-Related Gene Expression

The expression of stress- (*rpoS, oxyR*, and *soxR*) and virulence- (*stx1* and *stx2*) related genes in *E. coli* O157:H7 in response to the simulated microgravity was evaluated using qRT-PCR (primer sequences are given in Supplementary Table S1). Each 25 μl reaction contained 2 μl reverse-transcribed cDNA, 12.5 μl Maxima SYBR Green/ROX qPCR master mix (Thermo Scientific, Hampton, NH, United States), 0.2 μM of each primer, and 5.5 μl nuclease-free water. The Maxima SYBR Green/ROX qPCR master mix contained the following components: Maxima^®^ Hot Start Taq DNA polymerase, dNTPs, SYBR^®^ Green I dye, ROX passive reference dye, and optimized PCR buffer. The qRT-PCR was carried out using a Bio-Rad iQ5 thermal cycler (Bio-Rad), with the following conditions: pretreatment at 50°C for 2 min; an initial denaturation at 95°C for 10 min; and 40 cycles of denaturation, annealing, and extension, i.e., 95°C for 15 s, 63°C for 1 min, and 72°C for 30 s, respectively. The fluorescence data were collected at the end of each cycle. No template controls were included and consistently no Ct values were obtained for any of the negative controls (data not shown). The relative gene expression was determined using the comparative critical threshold (2^-ΔΔCT^) method (Schmittgen and Livak, 2008). The expression of the glyceraldehyde-3-phosphate dehydrogenase (GAPDH) gene was used to normalize the input amounts of RNA and the expression of the target genes was then determined. All experiments were conducted six times.

### Time-Kill Studies

Fresh MH broth containing antibiotic concentrations corresponding to 4 × MIC was inoculated with an overnight culture (final cell density of ca. 5 × 10^5^ CFU/ml) and incubated under LSMMG and NG, as described above. Samples were removed through the port in the HARV over the course of 48 h (0, 3, 6, 12, 24, and 48 h). The zero headspace was maintained, and the cell viability was determined as described above. Control samples containing MH broth with no antibiotics were prepared and tested in the same way. All experiments were repeated six times.

### Flow Cytometry

For the flow cytometry analysis, 24 h LSMMG and NG cultures grown in the presence of 4 × MIC of each antibiotic were diluted or concentrated to obtain 10^7^ cells/ml in filtered (pore size, 0.22 μm) phosphate-buffered saline (PBS). The cells were washed twice and diluted in PBS supplemented with 2 mg/L AFH (absorption: 630 nm, emission: 645 nm; Invitrogen, Carlsbad, CA, United States). They were then incubated at room temperature for 15 min in the dark, and washed with PBS before analysis.

Flow cytometry analysis was performed using a FACS Caliber flow cytometer (Becton Dickinson Biosciences, San Jose, CA, United States) with an excitation wavelength of 635 nm (the red diode). An FSC/SSC (side scattered light) diagram was used for gating to define the target bacterial cells. The data were analyzed using the BD CellQuest Pro software (Becton Dickinson Biosciences). An appropriate optical filter (FL4) was used to measure the fluorescence emitted by AFH. The signals were detected by a photodiode detector with a forward scatter voltage setting of E02, and 10,000 events were collected for each sample. The acquired events were presented as a histogram, with the M1 region set using live and dead cells, and the percentage of cells in the M1 region was then calculated.

### Modified EtBr Accumulation Assay

Accumulation of EtBr was evaluated as described previously (Smith and Blair, 2013), with the following modifications to determine the effect of LSMMG over a longer incubation period of at least 24 h. The cultures were grown to an OD_600_ of 0.6 (mid-exponential phase) and then washed three times with PBS. For optimum efflux conditions, glucose was added to a final concentration of 0.4% in PBS, and, where it was required to negate the efflux of EtBr in the absence of glucose, 100 μM carbonyl cyanide m-chlorophenylhydrazone (CCCP) was used. Thereafter, EtBr was added to a final concentration of 5 μg/ml and the bacterial cultures were incubated at 37°C (optimum) and 25°C (no activation) for 24 h in the HARV-RCCS, as described above. Following incubation, 200 μl of each culture was transferred to the wells of a black microtiter tray (Thermo Fisher Scientific, Rochester, NY, United States) and sample fluorescence was determined at the excitation and emission wavelengths of 535 and 620 nm, respectively, using a Hidex Sense plate reader (HIDEX, Turku, Finland). The measurements were recorded at least six times.

### Phase-Contrast Microscopy

Microscopic observations were conducted using samples grown for 24 h in broth containing 4 × MICs of antibiotics. After centrifugation (3000 × *g* for 15 min at 4°C) (MF80, Hanil Science Industrial, Co., Ltd., Gangneung, South Korea), the pellet was resuspended in PBS and 5 μl of the cell suspension was deposited onto a glass slide to be observed using phase-contrast microscopy (Leica DM6000, Leica Microsystems GmbH, Mannheim, Germany). Images were captured using a Leica DFC360 FX camera. Dimensions of each bacterial cell (*n* = 30 per group with cells randomly selected from 10 different fields of view) were measured by ImageJ software (U.S. National Institutes of Health, Bethesda, MD; United States^1^) and normalized according to the cell length (μm).

### Statistical Analysis

Statistical analysis was performed using SAS version 9.4 (SAS Institute, Inc., Cary, NC, United States). The data were evaluated using a general linear model for variance analysis. Tukey’s *t*-test was used to determine the significance of the differences in bacterial survival in *E. coli* O157:H7 cultures incubated under LSMMG and NG.

## Results

### Conventional Antibiotic Susceptibility Testing

**FIGURE 1:**
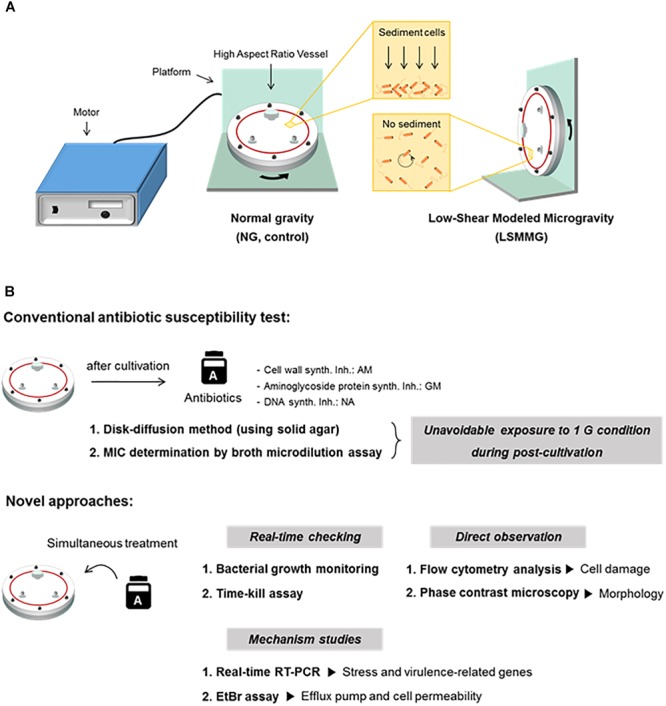
**(A)** The High-Aspect Ratio Vessel (HARV)-Rotary Cell Culture System used to generate simulated microgravity in ground-based investigations. **(B)** Schematic representations of a conventional antibiotic susceptibility test and novel approaches applied in the current study.

The susceptibility of *E. coli* O157:H7 ATCC 43895 cultivated under LSMMG (spaceflight analog conditions) and NG (Earth conditions) was first assessed against different antibiotics using the disk diffusion method (Figure 1 and Table 1). A comparison of bacterial antibiotic susceptibilities indicated no difference between cultures grown under NG and LSMMG conditions for all antibiotics, and the results indicated that the bacteria were equally susceptible. MICs determined using the broth microdilution method also indicated no difference in antibiotic susceptibility between LSMMG and NG cultures. The MICs against AM, GM, and NA were 1.0, 0.5, and 4.0 mg/L, respectively.

### Bacterial Growth in the Presence and Absence of Sub-inhibitory Antibiotic Concentrations

**FIGURE 2:**
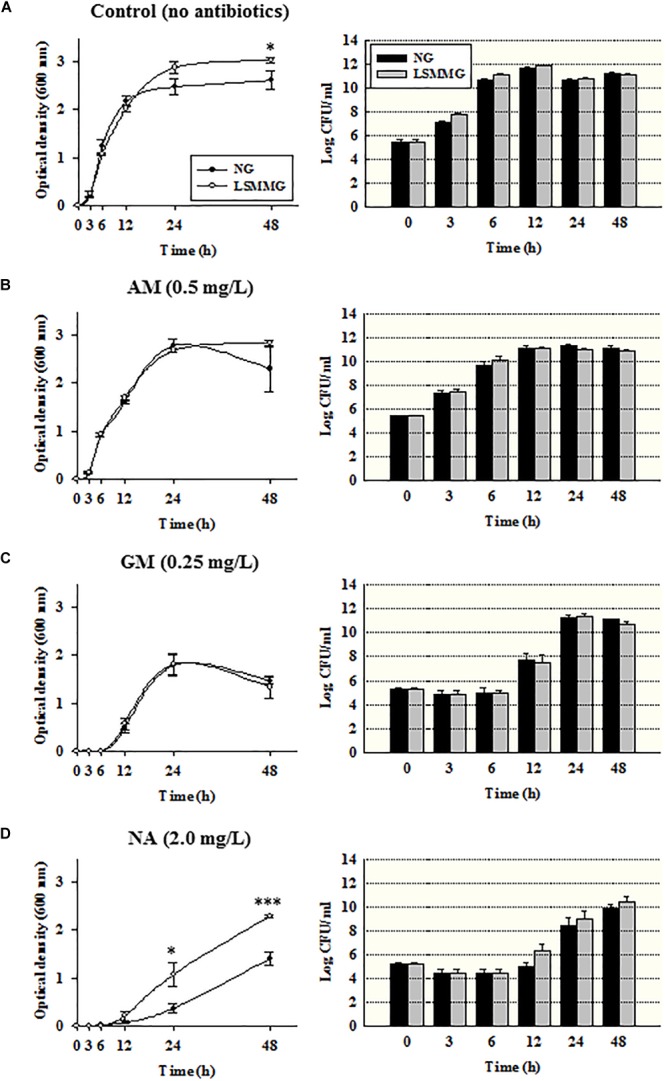
Optical density at 600 nm and viable cell counts of *Escherichia coli* O157:H7 cultured under NG and LSMMG in **(A)** MH broth with no antibiotics, or with the sub-inhibitory antibiotic concentrations (1/2 × MIC); **(B)** 0.50 mg/L of AM; **(C)** 0.25 mg/L of GM; and **(D)** 2.0 mg/L of NA. The data are presented as the mean ± standard error (SE) from 6 to 10 independent experiments. The asterisks represent significant differences between NG and LSMMG cultures (^∗^*P* < 0.05, ^∗∗^*P* < 0.01, and ^∗∗∗^*P* < 0.001).

The growth curves of bacteria cultured in MH broth in the presence or absence of 1/2 × MIC antibiotic concentrations were plotted as a function of the optical density at 600 nm (OD_600_) against time (Figure 2). The ODs of *E. coli* O157:H7 cultured for 48 h in MH broth only (antibiotic-free control) under different gravity conditions were significantly different (Figure 2A, LSMMG: OD_600_ 3.2; NG: OD_600_ 2.7; *P* < 0.05). However, when *E. coli* O157:H7 was cultivated with NA under LSMMG and NG, bacterial growth was significantly lower than in the controls and the growth patterns under different gravity conditions were also significantly different at a particular time (Figure 2D, *P* < 0.05). When cultivated with NA or GM (Figure 2C), the commencement of bacterial growth was not observed until after 6 h of incubation. ODs at 12 h were the lowest when grown in the presence of NA (LSMMG: OD_600_ 0.22; NG: _OD600_ 0.08) compared with the other treatments (LSMMG: OD_600_ 0.6–2.2; NG: OD_600_ 0.5–2.2). The OD_600_ of strain ATCC 43895 with NA was significantly higher under LSMMG than NG at 24 h (*P* < 0.05) and 48 h (*P* < 0.001). High optical densities in the presence of NA were noted after 48 h of culture under LSMMG (LSMMG: OD_600_ 2.3; NG: OD_600_ 1.4). Since no significant differences between LSMMG and NG cultures were apparent in the corresponding viable cell counts during bacterial growth (Figures 2A–D) differences in the optical density in the control and NA treated cultures under LSMMG were probably the result of changes in cell morphology (supportive observations of an increased cell size under LSMMG are presented in Supplementary Figure S1).

### Transcriptional Changes Elicited by Sub-inhibitory Concentrations of Antibiotics

**FIGURE 3:**
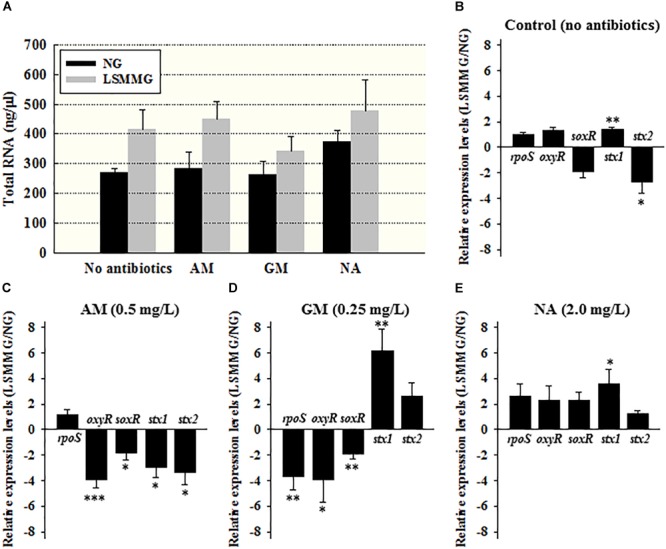
**(A)** Total RNA concentrations (ng/μl), and the average relative expression of the stress or virulence-related genes in *E. coli* ATCC 43895 cells cultured under NG and LSMMG in **(B)** MH broth (no antibiotics) and in the presence of 1/2 × MIC of **(C)** AM, **(D)** GM, and **(E)** NA for 24 h. The data are presented as the mean ± standard error (SE) from six independent experiments. The asterisks represent significant differences between NG and LSMMG cultures (^∗^*P* < 0.05, ^∗∗^*P* < 0.01, and ^∗∗∗^*P* < 0.001).

Alterations in the expression of genes in *E. coli* O157:H7 ATCC 43895 (24 h culture) were examined using the quantitative real-time polymerase chain reaction (qRT-PCR; the total RNA concentrations in the samples are shown in Figure 3A). The relative LSMMG/NG expression ratios of the stress-related genes (*rpoS* for general stress and *oxyR* and *soxR* for oxidative stress) and virulence-related genes (*stx1* and *stx2*) are shown in Figures 3B–E.

The expression of stress-related genes was not significantly changed (Figure 3B, *P* > 0.05). However, marked differences in gene expression were noted when the bacterial cells were cultivated in the presence of sub-inhibitory concentrations of antibiotics. The expression of all stress-related genes tested was reduced under LSMMG in the presence of 1/2 × MIC of AM (Figure 3C) and GM (Figure 3D), with average fold-changes of -1.8 to -3.9, and -1.9 to -3.9, respectively. The exception was *rpoS* expression under AM treatment (*P* > 0.05). For the NA treatment (Figure 3E), expression of *rpoS, oxyR*, and *soxR* tended to be up-regulated, but there was no significant difference (*P* > 0.05).

In the case of the toxin genes, the expression of *stx1* was up-regulated (1.3-fold, *P* < 0.01) and the expression of *stx2* was down-regulated (2.8-fold, *P* < 0.05) in MH broth in the absence of antibiotics under LSMMG (Figure 3B). When cultivated in the presence of AM, however, the expression of both toxin genes was significantly reduced under LSMMG conditions, from an average of 2.9- to 3.3-fold (Figure 3C, *P* < 0.05). The GM and NA treatments induced significant increase of *stx1* expression under LSMMG conditions from 3.5- to 6.2-fold (Figures 3D,E), while there was no significant difference in the expression of *stx2* (*P* > 0.05).

### Time-Kill Assay Under LSMMG

**FIGURE 4:**
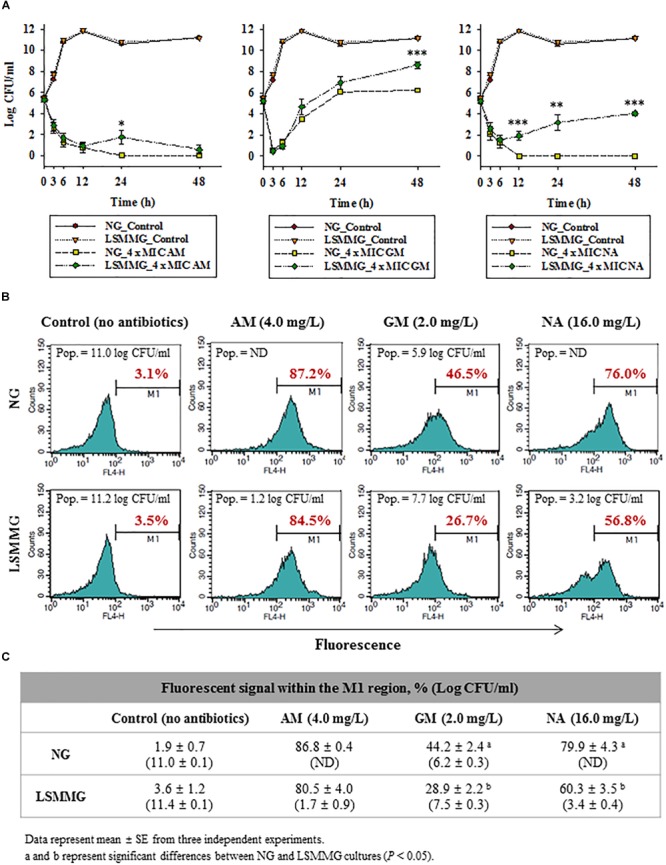
**(A)** Time-kill curve analysis of AM, GM, and NA under NG and LSMMG for *E. coli* O157 ATCC 43895 in MH broth. 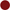, growth control under NG; 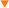, growth control under LSMMG; 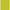, antibiotics at 1/2 × MIC under NG; 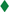, antibiotics at 1/2 × MIC under LSMMG; 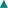, antibiotics at 4 × MIC under NG; 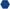, antibiotics at 4 × MIC under LSMMG. The data are presented as the mean ± standard error (SE) from six independent experiments. The asterisks represent significant differences between NG and LSMMG cultures (^∗^*P* < 0.05, ^∗∗^*P* < 0.01, and ^∗∗∗^*P* < 0.001). **(B,C)** Flow cytometry analysis of *E. coli* O157:H7 ATCC 43895 treated, or not, with 4 × MIC of AM, GM, and NA under NG and LSMMG, for 24 h in MH broth: **(B)** histogram of fluorescent signal and **(C)** average fluorescent signal within the M1 region (%).

As shown in Figure 4A, rapid bactericidal effects were produced in the presence of all antibiotics at 4 × MIC after 3–6 h of exposure, but for the GM and NA treatments, bacterial regrowth was observed. When *E. coli* O157:H7 cells were treated with AM at 4 mg/L (4 × MIC) and incubated under NG, cell concentrations gradually decreased and no viable cells were detectable after 24 h. By contrast, AM did not completely abolish the viability of cells under LSMMG after 24 and 48 h incubations, with 1.7 and 0.6 log_10_ CFU/ml of cells surviving, respectively. GM was bactericidal at 2 mg/L after a 3 h incubation, but bacterial regrowth was observed after 3 h incubation under both gravity conditions. At the end of the incubation period (48 h), the microbial populations under LSMMG were significantly higher than under NG, with concentrations of 8.6 and 6.2 log_10_ CFU/ml, respectively (*P* < 0.001). NA was bactericidal at 4 × MIC (16 mg/L) after 3 and 6 h of incubation under both gravity conditions, and cells were no longer detectable from 12 h incubation under NG. However, cells cultivated under LSMMG grew after 6 h and showed significantly higher populations than those under NG (1.9 log_10_ CFU/ml at 12 h, *P* < 0.001; 3.2 log_10_ CFU/ml at 24 h, *P* < 0.01; 4.0 log_10_ CFU/ml at 48 h, *P* < 0.001).

### Flow Cytometry Analysis of Cellular Damages

To understand the increased bacterial resistance to antibiotics under LSMMG, cellular damage after exposure to antibiotics was evaluated using flow cytometric analysis with Alexa Fluor 633 hydrazide (AFH), a dye that targets protein carbonylation (Saint-Ruf et al., 2016).

Antibiotic-treated and untreated *E. coli* O157:H7 ATCC 43895 cells cultured under NG and LSMMG were stained with AFH, and the changes in the distributions of fluorescent signal intensity were compared. Results are shown in Figures 4B,C (mean values from three independent experiments). The histograms reveal that, when compared with untreated cells, the fluorescent signal emitted by antibiotic-treated cells shifted to the M1 region, i.e., the samples contained more damaged, or more precisely carbonylated, cells. The carbonylated cells within the population of untreated *E. coli* O157:H7 cells cultured under LSMMG and NG represented only 1.9–3.6% of the total population, but this proportion increased to 28.9–86.8% after a 24 h treatment with 4 × MIC of AM, GM, and NA. The AM treatment resulted in similar percentages of carbonylated cells under LSMMG and NG, with 80.5% for 1.7 log_10_ CFU/ml in the LSMMG culture and 86.8% for no detectable cells in the NG culture, respectively (no significant difference, *P* > 0.05). The GM and NA treatments, however, resulted in a lower degree of cell carbonylation under LSMMG than under NG. Only 28.9% of the GM-treated cells were damaged under LSMMG (7.5 log_10_ CFU/ml), while 44.2% of cells were damaged under NG (6.2 log_10_ CFU/ml) (*P* < 0.05). The percentage of damaged cells treated with NA under LSMMG was 60.3% (3.4 log_10_ CFU/ml), while that of cells under NG was 79.9% (ND, not detected) (*P* < 0.05).

### Efflux Pump Activity Under LSMMG

**FIGURE 5:**
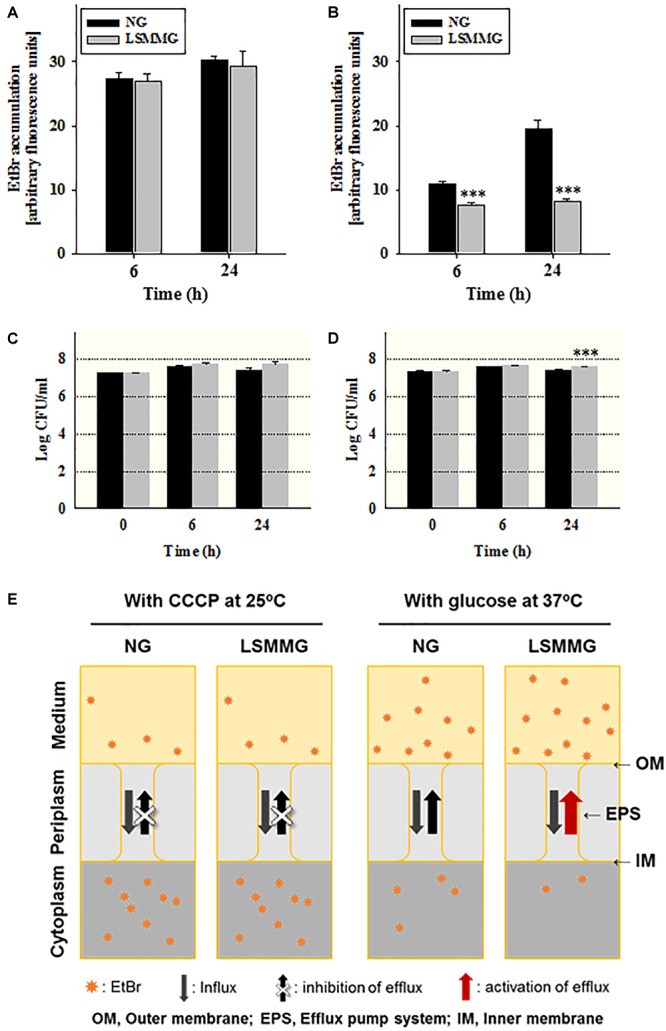
EtBr accumulation in *E. coli* O157:H7 ATCC 43895 cells cultured under NG and LSMMG for 24 h in **(A)** the presence of 100 μM CCCP at 25°C and **(B)** the presence of 0.4% glucose at 37°C. **(C,D)** Viable cell counts after each treatment from **(A,B)**, respectively. The data are presented as the mean ± standard error (SE) from six independent experiments. The asterisks represent significant differences between NG and LSMMG cultures (^∗^*P* < 0.05, ^∗∗^*P* < 0.01, and ^∗∗∗^*P* < 0.001). **(E)** A schematic representation of EtBr efflux under NG and LSMMG.

EtBr was used to examine the effect of LSMMG on efflux pump activity and cell permeability. The fluorescent signal of extracellular EtBr is very low, but it is amplified once it enters the cell. The EtBr concentration that resulted in the highest fluorescence with no significant reduction of the initial cell concentration was determined to be 5.0 mg/L (Supplementary Figure S2). The results of the EtBr accumulation assay under simulated microgravity are shown in Figure 5. Under conditions where efflux was inhibited (CCCP 100 μM, no glucose at 25°C), there was no significant difference in EtBr accumulation (measured using arbitrary fluorescence units) and cell populations between NG and LSMMG cultures (Figures 5A,C). The presence of 0.4% glucose in the medium resulted in the activation of the efflux pump at 37°C (optimal conditions), leading to a reduced accumulation of EtBr in both NG and LSMMG cultures (Figure 5B). The EtBr accumulation in cells under LSMMG was significantly lower than in cells under NG after a 6 h cultivation (NG: 10.8 units; LSMMG: 7.7 units; *P* < 0.001). The accumulation of EtBr increased, and the fluorescence of LSMMG cultures was lower than that of NG cultures (NG: 19.5; LSMMG: 8.2; *P* < 0.001), after prolonged exposure (24 h).

### Antibiotic-Elicited Morphological Changes

**FIGURE 6:**
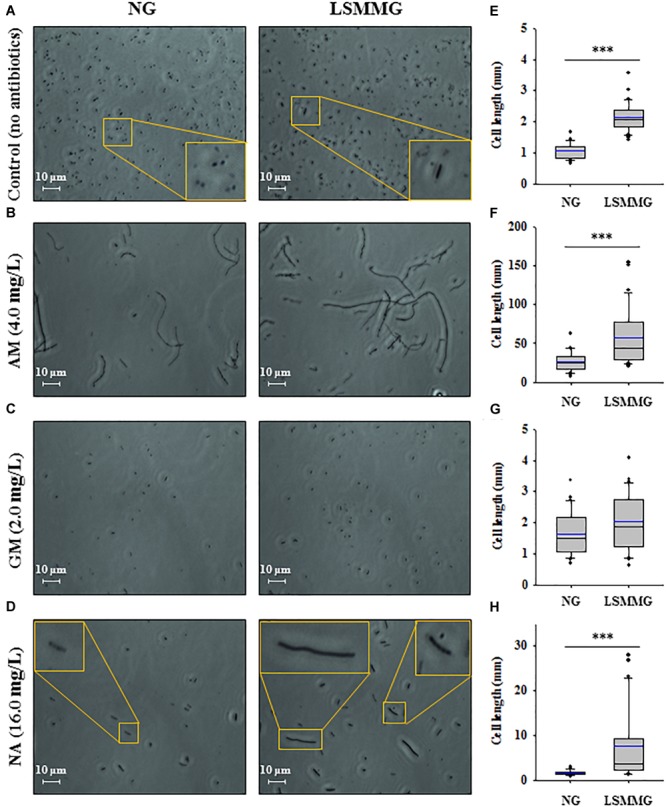
**(A–D)** The liquid cultures of strain ATCC 43895 in the absence (control) or presence of antibiotics (at 4 × MIC) were centrifuged to concentrate the residual biomass; the wet mount preparations were then analyzed using phase-contrast microscopy. Scale bar: 10 μm. **(E–H)** Cell lengths in bacterial cultures in **(A–D)**, respectively. The squares indicate the interquartile range for each data point, and the black and blue lines denote the median and mean values, respectively. The error bars above and below the squares indicate the 90^th^ and 10^th^ percentiles, respectively, and the black circles represent the outliers. The asterisks represent significant differences between NG and LSMMG cultures (^∗^*P* < 0.05 and ^∗∗∗^*P* < 0.001).

Flow cytometric analysis of *E. coli* O157:H7 cells cultured under LSMMG and NG revealed that there were differences between them in the forward scatter (FSC)/side scatter (SSC) dot plot (Supplementary Figure S3). This suggests morphological differences in the cells. To determine whether antibiotic treatment under LSMMG induced changes in bacterial morphology when compared with NG, the morphology of *E. coli* O157:H7 ATCC 43895 cells was examined in the absence (control) or presence of the selected antibiotics (Figure 6).

Bacterial cells cultured in control MH broth under LSMMG were medium/long rods, whereas their NG counterparts were shorter and oval-shaped. The average cell length of cells in LSMMG cultures was significantly greater than in NG cultures (Figure 6A, NG: 1.1 μm; LSMMG: 2.2 μm; *P* < 0.001). LSMMG cultures had higher FSC intensities than NG cultures with increased cell granularity (Supplementary Figure S3A, LSMMG: 14.8%, NG: 23.9% in the M2 region). Treating *E. coli* O157:H7 with 4 × MIC of AM caused the cells to become filamentous, and this effect was prominent in LSMMG cultures (Figure 6B, *P* < 0.001). With respect to the filamentous cell size (oval-shaped cells were excluded from measurements), the cell length in LSMMG cultures ranged from 20.5 to 153.9 μm (average: 57.4 μm), whereas cell length in NG cultures ranged from 7.7 to 43.6 μm (average: 27.5 μm). Increased cell length was also apparent from FACS analysis, with the FSC intensity of LSMMG cultures in the M2 region being higher than that of NG cultures (Supplementary Figure S3B, LSMMG: 24.4%, NG: 28.9%). However, in this case, oval-shaped cells were included from measurements. Treatment with GM did not induce cell filamentation since there was no significant difference in cell length between NG and LSMMG cultures (Figure 6C). NA treatment, however, induced morphological changes in cells grown under LSMMG and longer bacteria were frequently observed (Figure 6D, NG: 1.6 μm; LSMMG: 8.6 μm; *P* < 0.001).

## Discussion

Most antibiotic susceptibility studies with ground-based microgravity simulation performed to date have focused on measuring the MIC values for bacteria either in space (Tixador et al., 1985b) or after landing (Juergensmeyer et al., 1999). Although the MIC is the most widely used parameter for evaluating the efficacy of antibiotics, its main limitation is the lack of information on the rate of the antibacterial effect (Mueller et al., 2004). To test the antibiotic efficacy under microgravity more accurately on Earth, novel approaches to determine the activities of antibiotics are needed.

The antibiotic susceptibility of *E. coli* O157:H7 cells that had been cultivated under LSMMG was first tested using conventional disk diffusion and MIC determination methods (Table 1), which revealed no differences between the LSMMG and NG cultures. Previous studies have shown no alterations in antibiotic sensitivity of post-LSMMG cultures (Kacena and Todd, 1999; Tucker et al., 2007). However, some studies have reported that antibiotics are less effective in suspension cultures in comparison to solid agar surface cultures in the space environment, and that these traits are not retained following a spaceflight (Lapchine et al., 1986, 1987; Tixador et al., 1994). These previous reports support the current observations by conventional antibiotic susceptibility tests. The antibiotic susceptibility tests suggested that ground-based studies on antibiotic efficacy should be performed in liquid cultures under LSMMG conditions. The current study used alternative methods for the evaluation of antibiotic efficacy, i.e., a real-time growth-monitoring and a time-kill assay (Mueller et al., 2004), and their advantage over the standard MIC approach lies mainly in enabling time-dependent measurements in liquid culture under LSMMG (Figure 1).

Unlike the results of conventional antibiotic susceptibility testing, significant differences for some antibiotics were apparent in the time-dependent growth (Figure 2) and killing (Figure 4) data. Although bacterial growth (measured by optical density) was greater in LSMMG cultures than in NG cultures after 48 h in the antibiotic-free control samples (Figure 2A), no significant differences in growth were apparent between LSMMG and NG cultures exposed to 1/2 × MICs of AM and GM (Figures 2B,C, *P* > 0.05). By contrast, the optical densities of *E. coli* O157:H7 cultures were significantly higher under LSMMG than under NG after 24 and 48 h incubation in the presence of NA (Figure 2D, *P* < 0.05 and *P* < 0.001, respectively). The mechanisms behind these effects at sub-inhibitory antibiotic concentrations under LSMMG could be explained as follows: when bacteria are exposed to antibiotics during cell division, the drugs can readily bind to the target, leading to no significant differences in cell mass between LSMMG and NG cultures, but in the case of NA treatment, it is hypothesized that other genetic differences explain the observations.

The bacterial general stress response, regulated by σ^s^ (the product of the *rpoS* gene), is responsible for bacterial resistance to antibiotics because it results in the expression of core proteins that protect such biomolecules as the cell wall, proteins, and DNA (Matin, 2009). As bactericidal antibiotics damage these molecules, σ^s^ is likely to have played a role in protecting bacteria against the antibiotics used here. Indeed, a previous study reported increased translational efficiency of *rpoS* in generic *E. coli* under LSMMG conditions (Lynch et al., 2004). Bactericidal antibiotics also induce the production of reactive oxygen species (ROS) by activating the tricarboxylic acid cycle (Lobritz et al., 2015). The *oxyR* and *soxR* genes are oxidative stress-related genes that counter the challenges posed by ROS (Lushchak, 2001). These stress-related genes were all down-regulated (1.8- to 3.9-fold) in LSMMG cultures cultivated with 1/2 × MIC AM and GM showing no significant difference in optical densities when compared to corresponding NG cultures, except for *rpoS* expression in AM treatment (*P* > 0.05; Figures 3C,D). In the case of NA treatment, however, *rpoS, oxyR*, and *soxR* tended to be up-regulated (3.1- and 2.7-fold, respectively), which supports the observation of bacterial growth in the presence of sub-inhibitory concentrations of NA under LSMMG.

Previous studies have reported increased virulence of pathogenic *E. coli* under LSMMG, for example, increased intimin production in enterohemorrhagic *E. coli*, increased expression of heat labile enterotoxin in enterotoxigenic *E. coli*, and increased adherence of adherent-invasive *E. coli* (Carvalho et al., 2005; Chopra et al., 2006; Allen et al., 2008). The ability to produce Shiga toxin, encoded by the *stx* genes, is the key virulence trait of *E. coli* O157:H7. Here, *stx1* was up-regulated while *stx2* was down-regulated in bacterial cells cultured in the absence of antibiotics under LSMMG (Figure 3B). Unlike the AM treatment, exposure to GM induced the up-regulation of *stx1* (6.2-fold, *P* < 0.01) and NA also induced the up-regulation of *stx1* (3.5-fold, *P* < 0.05), suggesting that GM and NA might not be appropriate therapeutic choices against this strain of *E. coli* O157:H7 under microgravity (Figures 3C–E). Since the current results have identified potential risks in using inappropriate antibiotics against the Shiga toxin-producing *E. coli* O157:H7 during spaceflight, a thorough examination of antibiotic use (i.e., their type, amounts, side effects, etc.) in space should be conducted both *in vitro* and *in vivo*.

Unlike the observations of bacterial growth in the presence of sub-inhibitory antibiotic concentrations, the time-kill assay revealed a significantly higher viability of LSMMG cultured cells than NG cultured cells in the presence of the tested antibiotics, i.e., a reduced antibiotic efficacy under LSMMG (Figure 4). Viable cell counts were higher under LSMMG than under NG in response to 4 × MIC of AM and GM at 24 h (*P* < 0.05) and 48 h (*P* < 0.001), respectively (Figure 4A). These trends were more obvious during exposure to NA where regrowth was observed under LSMMG after 6 h, while no bacteria were detected from 12 h onwards under NG.

To gain further insight into the mechanism of the increased bacterial resistance to antibiotics under the simulated microgravity, the degree of cellular damage after exposure to antibiotics was evaluated by flow cytometry using AFH, a dye that targets protein carbonylation (Saint-Ruf et al., 2016). Antibiotic efficacy is linked to bacterial cellular respiration (Lobritz et al., 2015). ROS generated by antibiotics damages the proteins in bacterial cells by, for example, irreversible protein carbonylation. Thus, determination of the extent of carbonylation of antibiotic-treated cells can be used as a marker of impaired cell viability and an indirect measure of ROS generation. The degree of protein carbonylation elicited in *E. coli* O157:H7 ATCC 43895 cells by GM and NA treatment was lower under LSMMG than under NG (Figures 4B,C). Treatment with AM, resulting in small differences in viable cell counts under LSMMG and NG, also resulted in small differences in the degree of carbonylation (*P* > 0.05). This might be because the main target of AM is the cell wall, the outermost protection system of bacteria, while GM and NA have to penetrate the cell envelopes to reach their targets in the cytoplasm. It is hypothesized that the concentration of AM molecules at the cell wall might not be significantly different between LSMMG and NG cultures. However, the degree of carbonylation, and hence damage, associated with GM and NA treatments under LSMMG was significantly lower than under NG.

Penetration and efflux of antibiotics are a significant matter for antibiotic efficacy (Miller, 2016). The possibility that an efflux pump is activated under simulated microgravity conditions resulting in an increased efflux of antibiotic molecules was investigated by measuring the accumulation of EtBr and its extrusion from *E. coli* O157:H7 cells, a result of the balance between the molecule’s entry (influx) and the extruding activity of the efflux pump (Viveiros et al., 2007). To test the permeability of bacterial cells under LSMMG and NG, EtBr accumulation was observed under conditions inhibitory for efflux (i.e., in the presence of CCCP). There was no significant difference in EtBr accumulation between LSMMG and NG cultures (Figure 5A, *P* > 0.05), indicating that efflux-independent cell permeability was not affected under LSMMG. However, the fluorescent intensity of the LSMMG culture was significantly lower than that of the NG culture (about twofold) in the presence of 0.4% glucose after 24 h, suggesting that less EtBr accumulated inside the cells because of a more activate efflux pump under LSMMG (Figures 5B,E). A previous report presented results demonstrating that the expression of efflux pump genes (*acrAB* and *tolC*) is up-regulated in *E. coli* ATCC 25922 under simulated microgravity (Xu et al., 2015).

It was also found that the growth of *E. coli* O157:H7 in a medium containing 4 × MIC of AM, a β-lactam antibiotic, under LSMMG resulted in more pronounced cell filamentation and septation than under NG (Figure 6). Various stresses, including exposure to β-lactam antibiotics, DNA damage, and high hydrostatic pressure (Pratt et al., 2012; Yadavalli et al., 2016), cause filamentation of *E. coli*. Although the protective effect of cellular filamentation in the context of antibiotic tolerance has not been established, some evidence exists that filamentation improves cell survival under various stresses. The control of cell division by a cell, as observed by us in the form of a division block, is generally assumed to enhance cell fitness under stress (Yadavalli et al., 2016). Though the cell damages measured by AFH were not significantly different between LSMMG and NG cultures in the current study, the degree of carbonylation of the cells tended to be reduced in LSMMG cultures (Figure 4B). This supports the significantly higher populations of LSMMG cultures at 24 h in the presence of AM (Figure 4A, *P* < 0.05). Taken together, these observations suggest that *E. coli* O157:H7 cells may try to maximize the chance of survival in the presence of antibiotics in response to LSMMG.

Here, an extensive analysis of *in vitro* antibiotic efficacy against Shiga toxin-producing *E. coli* O157:H7 cells under simulated microgravity was conducted to explore potential undesirable pharmacological effects during spaceflight. Of the three different antibiotics tested, sub-inhibitory concentrations of NA induced more active growth in LSMMG cultures than in NG cultures, with an accompanying increased expression of Shiga toxin genes. Previous studies have reported the virulence and susceptibility to antibiotics of bacteria under microgravity (Nickerson et al., 2000; Lawal et al., 2013), but, to the best of our knowledge, this is the first report describing the possibility that exposure of *E. coli* O157:H7 to inappropriate antibiotics could induce toxin production. Bactericidal effects of antibiotics were reduced under LSMMG, with decreased protein carbonylation of cells, suggesting that reduced ROS generation and increased filamentation enhanced cell fitness and counteracted antibiotic stress. Since efflux pumps are thought to play important roles in antibiotic resistance, efflux pump inhibitors might be considered to be useful for treating bacterial infections, but further studies evaluating their effects are needed. This paper presents novel approaches for studying antimicrobial efficacy under simulated microgravity, and the results could help choose the effective therapeutic choices during a space mission.

## Author Contributions

HK and MR conceived the experiments. HK performed experiments, analyzed the data, and wrote the manuscript. MR supervised all research.

## Conflict of Interest Statement

The authors declare that the research was conducted in the absence of any commercial or financial relationships that could be construed as a potential conflict of interest.

## Footnotes

1 http://rsbweb.nih.gov/ij/
