# Supplementary material for: Novel Antibiotic Testing Approaches Reveal Reduced Antibiotic Efficacy Against Shiga Toxin-Producing Escherichia coli O157:H7 Under Simulated Microgravity
Source: Front Microbiol. 2018 Dec 21;9:3214. doi: 10.3389/fmicb.2018.03214 (PMC6308135; doi:10.3389/fmicb.2018.03214)
Supplement: Supplementary file 1 [file Presentation_1.PDF]

[Supplementary Information]

Title: Novel Antibiotic Testing Approaches Reveal Reduced Antibiotic Efficacy against Shiga Toxin-Producing *Escherichia coli* O157:H7 under Simulated Microgravity

Authors: H.W. Kim and M.S. Rhee

1. Graphical abstract

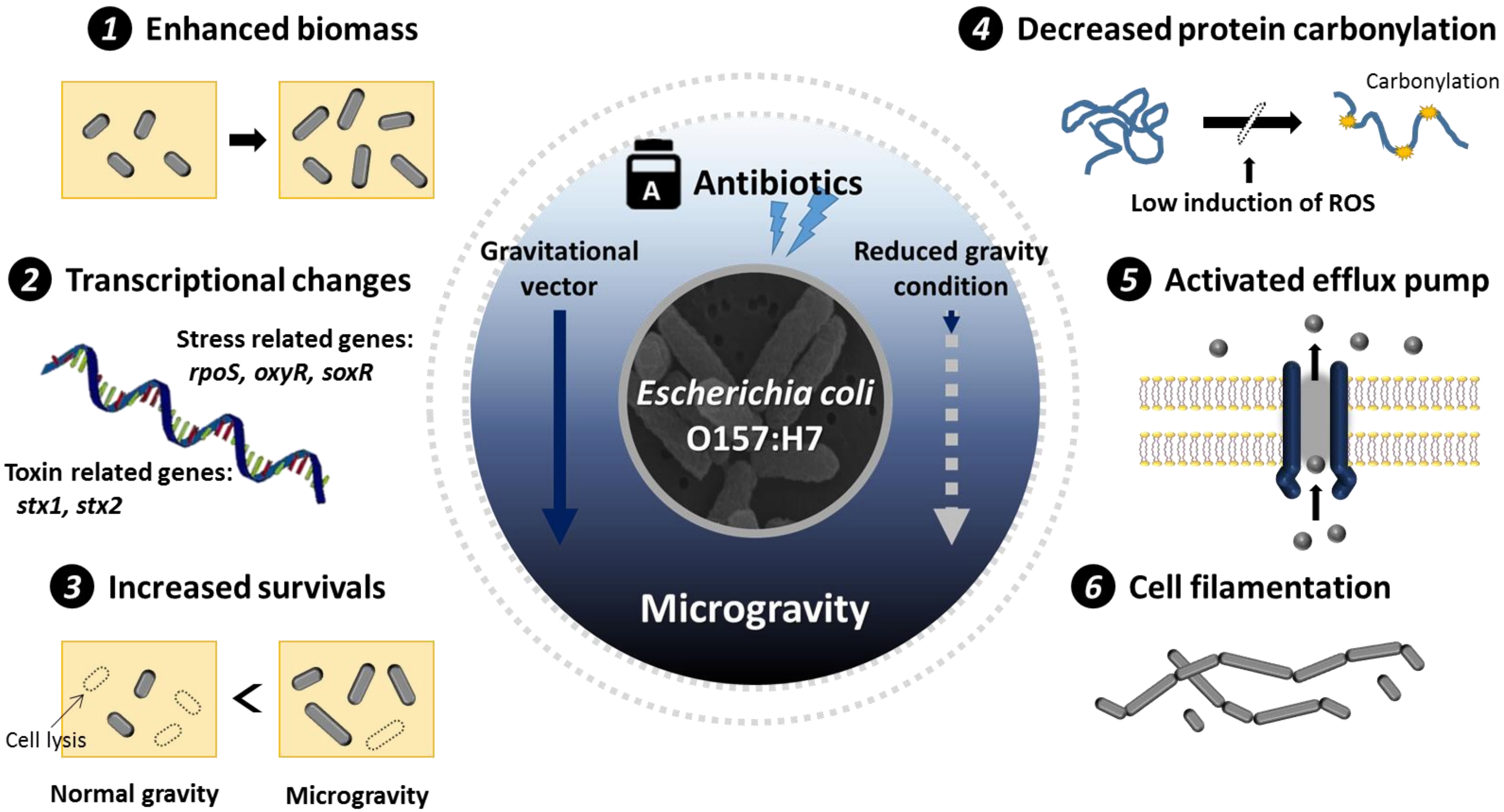

2. Supplementary table

Table S1. Primer Sequences Used in Real-Time RT-PCR

Function	Target gene	Sequence (5′ to 3′)	Reference
Housekeeping gene	GAPDH	For: TCC GTG CTG CTC AGA AAC G	Carey et al. (2009)
		Rev: CAC TTT CTT CGC ACC AGC G	
Stress-related gene	*rpoS*	For: CGC CGG ATG ATC GAG AGT AA	
		Rev: GAG GCC AAT TTC ACG ACC TA	
	oxyR	For: GAA GCA CAG ACC CAC CAG TT	Wang et al. (2009)
		Rev: CAA ACA ACG GCA CTT CAA TG	
	soxR	For: GCA TTA AAG CGC TGC TAA CC	
		Rev: ATT GCC GCT GTT ACG GAT AC	
Virulence-related gene	*stx1*	For: ATA AAT CGC CAT TCG TTG ACT AC	Paton and Paton (1998)
		Rev: AGA ACG CCC ACT GAG ATC ATC	
	stx2	For: GGC ACT GTC TGA AAC TGC TCC	
		Rev: TCG CCA GTT ATC TGA CAT TCT G	

3. Supplementary figures

Figure S1. Bacterial cell morphology under (A) NG and (B) LSMMG. The cells were grown in the absence of antibiotics for 24 h, and then visualized by transmission electron microscopy. Scale bars, 5 and 0.5 μm.

Figure S2. (A) The EtBr accumulation in *E. coli* O157:H7 ATCC 43895 cells significantly increased with an increasing EtBr concentration (mg/L) ($P < 0.05$). (B) The viable cell counts (log CFU/ml) decreased with an increasing EtBr concentration ($P < 0.05$). The data are presented as the mean ± standard error (SE) from six independent experiments. a to e, data are significantly different at the level of $P < 0.05$.

Figure S3. Flow cytometry analysis of *E. coli* O157:H7 treated, or not, with 4 × MIC of AM under NG and LSMMG, for 24 h in MH medium: FSC/SSC dot plot, histogram of FSC intensity, and histogram of SSC intensity of (A) control (no antibiotics) and (B) AM (4.0 mg/L) treated cells.

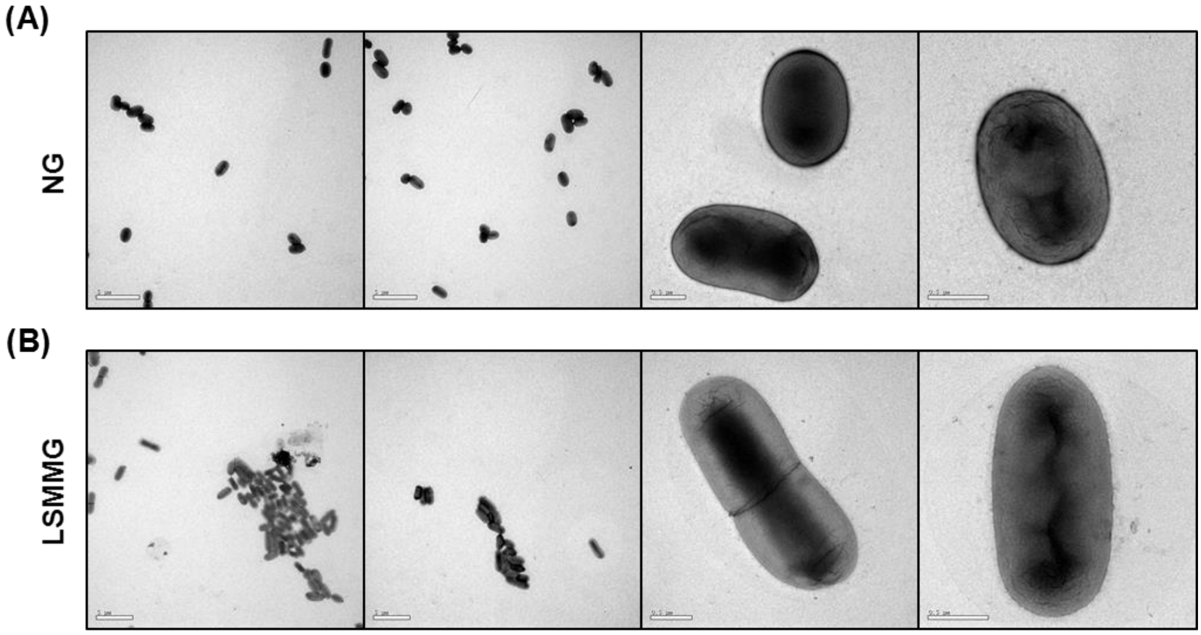

Figure S1.

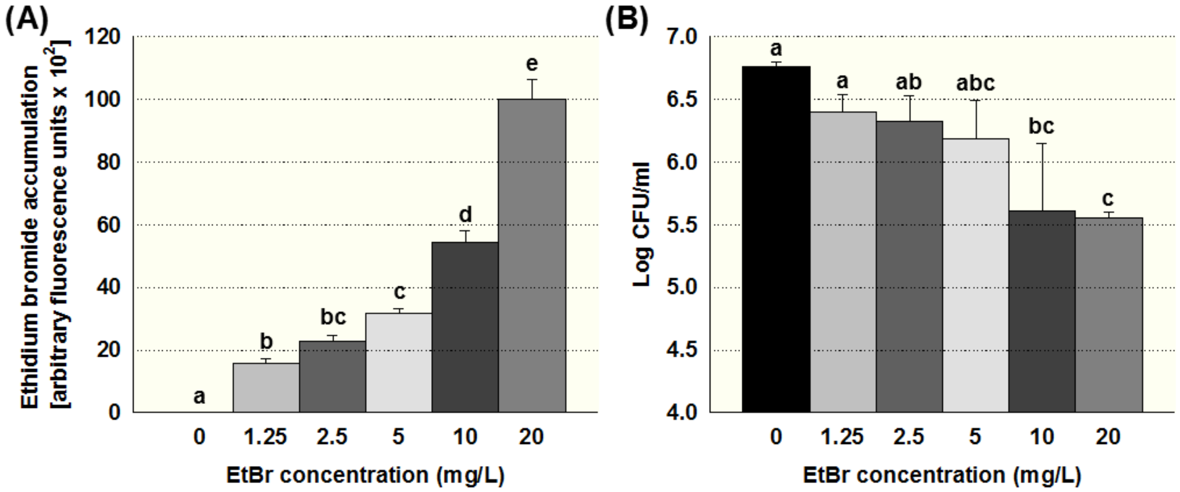
(A)
Ethidium bromide accumulation [arbitrary fluorescence units x 10²]
120
100
80
60
40
20
0
a
b
bc
c
d
e
0
1.25
2.5
5
10
20
EtBr concentration (mg/L)
(B)
Log CFU/ml
7.0
6.5
6.0
5.5
5.0
4.5
4.0
a
a
ab
abc
bc
c
0
1.25
2.5
5
10
20
EtBr concentration (mg/L)

Figure S2.

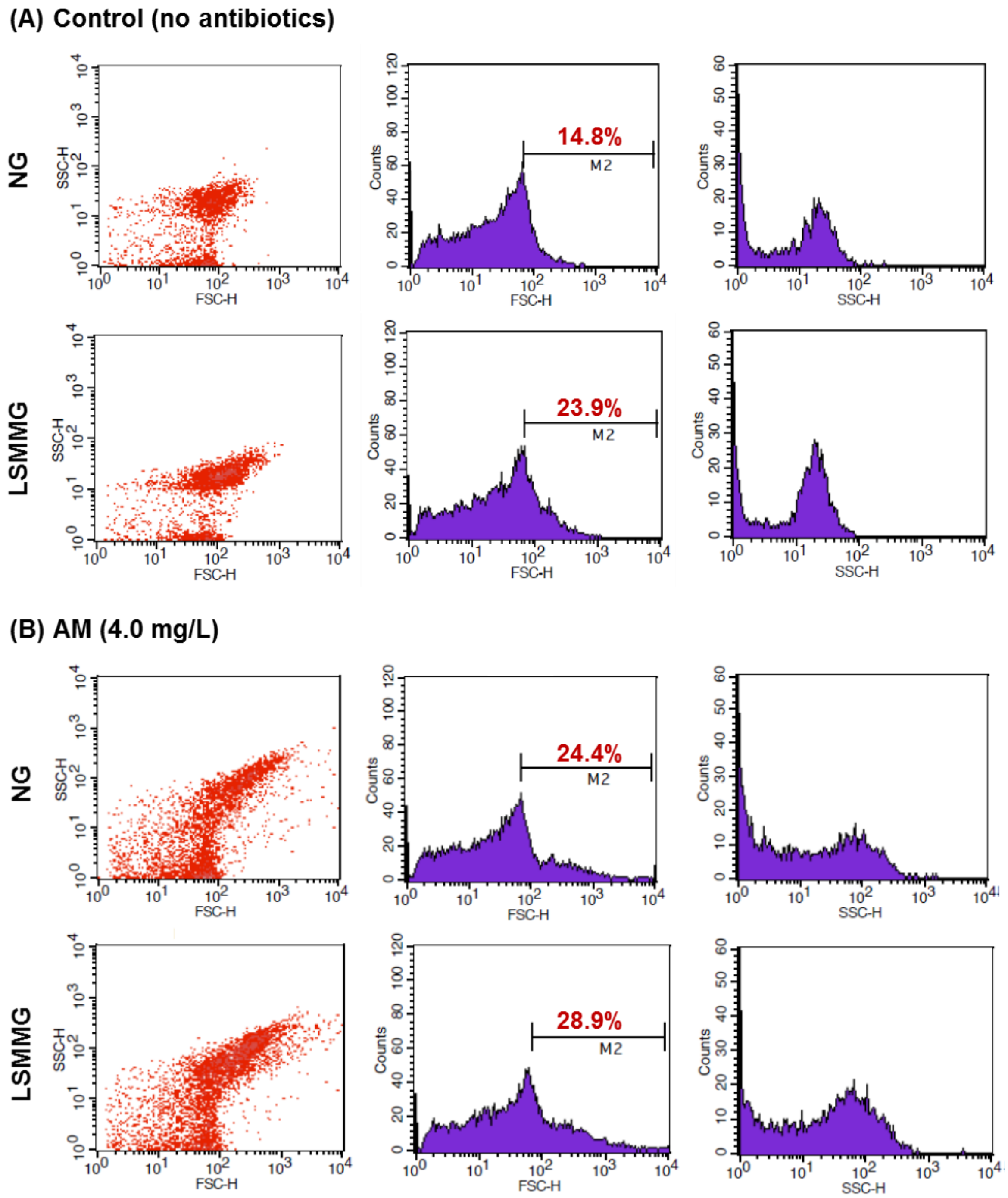
(A) Control (no antibiotics)
NG
LSMMG
14.8%
M2
23.9%
M2
(B) AM (4.0 mg/L)
NG
LSMMG
24.4%
M2
28.9%
M2
FSC-H
SSC-H
Counts

Figure S3.

4. Supplementary references

Carey, C.M., Kostrzynska, M., and Thompson, S. (2009). *Escherichia coli* O157: H7 stress and virulence gene expression on Romaine lettuce using comparative real-time PCR. *J. Microbiol. Meth.* 77**,** 235-242.

Paton, A.W., and Paton, J.C. (1998). Detection and Characterization of Shiga Toxigenic *Escherichia coli* by Using Multiplex PCR Assays for *stx 1*, *stx 2, eaeA*, Enterohemorrhagic *E. coli hlyA*, *rfb* O111, and *rfb* O157. *J. Clin. Microbiol.* 36**,** 598-602.

Wang, S., Deng, K., Zaremba, S., Deng, X., Lin, C., Wang, Q., Tortorello, M.L., and Zhang, W. (2009). Transcriptomic response of *Escherichia coli* O157: H7 to oxidative stress. *Appl. Environ. Microbiol.* 75**,** 6110-6123.
